# Superovulation Induced Changes of Lipid Metabolism in Ovaries and Embryos and Its Probable Mechanism

**DOI:** 10.1371/journal.pone.0132638

**Published:** 2015-07-13

**Authors:** Li-Ya Wang, Ning Wang, Fang Le, Lei Li, Hang-Ying Lou, Xiao-Zhen Liu, Ying-Ming Zheng, Ye-Qing Qian, Yun-Long Chen, Xin-Hang Jiang, He-Feng Huang, Fan Jin

**Affiliations:** 1 Centre of Reproductive Medicine, Women’s Hospital, School of Medicine, Zhejiang University, Hangzhou, 310006, China; 2 Key Laboratory of Reproductive Genetics (Zhejiang), Ministry of Health, Hangzhou, 310006, China; 3 Department of Gynecologic Oncology, Henan Cancer Hospital, Zhengzhou, 450003, China; 4 College of Life Science, Zhejiang University, Hangzhou, 310058, China; 5 International Peace Maternity and Child Health Hospital, Shanghai Jiao Tong University School of Medicine, 910 Hengshan Road, Shanghai, 200030, China; Institute of Zoology, Chinese Academy of Sciences, CHINA

## Abstract

This research was intended to investigate the fetal origins of changed birth weight of the offspring born through assisted reproductive technology (ART). The association between hormone and lipid metabolism or body weight has been generally accepted, and as the basic and specific treatment in ART procedure, gonadotropin stimulation might have potential effects on intrauterine lipid metabolism. In our studies, the mice were superovulated with two doses of gonadotropin. The cholesterol metabolism in ovaries and the triglyceride metabolism in embryos were analyzed. The results showed gonadotropin probably accelerated luteinization and induced a longer time follicle development and ovulation, which resulted in histological and morphological alteration of ovary, and increased the cholesterol content and the expressions of steroidogenesis-related genes. In embryos, gonadotropin increased lipid accumulation and decreased fatty acid synthesis in a dose-dependent manner. Moreover, the changes of fatty acid composition were also shown in superovulation groups. Our studies firstly provided the evidence that the superovulation might affect the maternal and fetal lipid metabolism. These variations of lipid metabolism in our results may be associated with birth weight of ART infants.

## Introduction

Previous studies have found infants whose mothers had received fertility treatment were at a high risk of abnormal birth weight [1–5]. Early “catch-up” growth is associated with postnatal growth patterns [6]. Epidemiological studies showed that low birth weight, either from low gestational age or slow prenatal growth or a combination of them, had increased risks of metabolic diseases, such as type 2 diabetes, obesity, hypertension, and cardiovascular diseases [7–10]. In 1990, Barker propounded the hypothesis “The fetal and infant origins of adult disease”, which pointed out that adverse intrauterine environment was a main factor disturbing fetal development [11–13].

Hormone influences body weight by regulating lipid metabolism and body fat distribution. Gonadotropin stimulation is the basic treatment for superovulation induction in assisted reproductive technology (ART). In women, the lipid metabolism changes with periodical shifted gonadotropin levels [14]. Hormone replacement therapy can be used to improve the abnormal lipid metabolism in postmenopausal women through increasing the expression of estrogen receptor [15–17]. Although estrogen and its receptor are related with lipid metabolism, their functions aren’t directly required for oocyte maturation and embryo development [18,19]. Follicle-Stimulating Hormone (FSH) and luteotropic hormone (LH) promote oocyte maturation and ovulation. The effect of hormones is based on the combined hormone-receptor complex. During follicle development, the levels of FSH receptor (FSHR) and LH receptor (LHCGR) can be regulated by pregnant mare serum gonadotropin (PMSG) and human chorionic gonadotropin (hCG). PMSG/FSH stimulation increases the mRNA expressions of *Fshr* and *Lhcgr* [20–23], and the subsequent hCG/LH administration decreases the expression of *Fshr* and *Lhcgr* [24]. During the entire estrous cycle, the ovarian follicle development and luteinization are the main processes that are closely related to lipid metabolism. Fatty acids provided energy for oocyte maturation and early embryo development [25,26]. The consumption and accumulation of ovarian cholesterol occur during ovulation and luteinization [27,28]. Gonadotropin can regulate steroidogenesis and *de novo* fatty acid synthesis [29]. Therefore, the interruption of normal estrous cycle might influence the lipid metabolism of ovaries and embryos.

PMSG-hCG protocol is commonly used for superovulation in animal experiment. But owing to their long half-life, the remaining hormone could last for a few days after injection. Our previous studies have found high birth weight of ART conceived mice (Fan J, unpublished data). How does the gonadotropin influence fetal lipid metabolism? Our research was intended to investigate the early influence of superovulation on maternal and fetal lipid metabolism. The results would provide a new evidence for the gonadotropin-induced lipid metabolism alteration during early embryo development.

## Materials and Methods

### Oocyte and embryo collection

All the protocols used in our investigation were approved by the Animal Care Ethics Committee of Zhejiang University. ICR female mice (6-8weeks) were divided into three groups: naturally conceived (NC), controlled ovarian hyperstimulation with low dose of gonadotropin (LCOH) and with high dose of gonadotropin (HCOH). There were 30 mice in each group. All mice were raised under an environment with room temperature 23±1°C, humidity 55±5% and a 12h light-dark cycle. Mice in LCOH group were injected by 5 IU PMSG (GEN’s, Hangzhou, China) and 5 IU hCG (GEN’s, Hangzhou, China) 48h later. The dosage of PMSG and hCG used for the mice in HCOH group was 10IU. The mice were sacrificed by neck breaking method. Metaphase II (MII) oocytes in COH groups were obtained 15h after hCG injection. MII oocytes in NC group were got from mice at estrus stage. After superovulation, the female mice were naturally mated with ICR male mice (10–12 weeks), and NC, LCOH and HCOH embryos at 2-cell stage were obtained from pregnant mice (the appearance of a vaginal plug was designated as 0.5 days) at 1.5 day (39h after hCG injection for COH groups).

### Collection and analyses of vaginal secretion

To decide the stage of estrous cycle of mice, the vaginal secretion was collected with the pasteur pipettes filled with 10μl 0.9%saline. Place the pipette into the vagina and flush the vagina with saline solution. The vaginal fluid was collected and placed on a glass slide. When the fluid was dry, it was fixed with 95% alcohol and stained with eosin (Sangon, Shanghai, China) for 15min. The determination of the estrous cycle phase is based on the proportion of the nucleated epithelial cells, anucleated cornified cells and leukocytes.

### Lipid staining

Nile red powder (Sigma) was dissolved in DMSO at 5mg/ml as stock solution. Embryos were fixed in 4% paraformaldehyde for 24h, and then 15 embryos of each group was washed in 1×PBS, 3 times, 5min each. The embryos were stained in Nile red with the final concentration of 10μg/ml for 5min, followed by washing in 1×PBS, 3 times, 5min each. All embryos were suspended in 40μg/ml DAPI (Sigma) for 5min and were observed in a cell culture dish with glass bottom (0.13–0.16mm). Images were acquired using Zeiss LSM 510 Meta confocal microscope. The red fluorescent signal was detected on 543nm excitation laser line using the same pinhole.

### Morphological analysis

All ovaries were obtained from the mice after oocyte and embryo collection. The female mice and their ovaries were weighed for ovarian weight index calculation. 5 ovaries in each group were fixed in 4% paraformaldehyde and embedded by wax. Every 10^th^ section per 5μm across the whole ovary was stained with hematoxylin and eosin. Secondary and antral follicles were estimated by exact counts on an Oylmpus BX51 microscope.

### Progesterone measurement

Blood samples were obtained from mouse eyes using a serum separator tube and serum was isolated through centrifugation. Progesterone levels were measured using a Mouse Progesterone ELISA kit (Cusabio Biotech., Wuhan, Hubei, China). Measurements were performed according to the manufacturer’s instructions.

### Cholesterol isolation and measurement

Ovaries were weighted and put into a tube with 1ml chloroform-methanol (2:1, V/V) solution. After thoroughly homogenized, the substances were removed by centrifugation and the fluid was dried using nitrogen gas (N_2_). To get total cholesterol, 1U cholesterol esterase (Worthington) were added and the reaction was proceeded at 37°C for 1 hour. 1ml hexane was used to extract the total lipid. The hexane phase was dried and re-dissolved in 50μl acrylonitrile- isopropanol (4:1). 20μl fluid was used to detect total cholesterol content. Standard curve were done by accurate weighting and gradient diluting the standard cholesterol (Sigma). All of the HPLC analyses used an Agilent 1200 system. The reactions were analyzed using an Agilent Extend-C18 column (5um, 4.6×250mm) thermostatted at 35°C, a solvent system of methanol: Acetonitrile (9:1, v/v) and a flow rate of 0.8ml/min. The mass detection was carried out by a Thermo Finnigan LCQ Deca XP MAX. The source type was Atmospheric Pressure Chemical Ionization (APCI) in positive polarity mode. The capillary temperature and APCI vaporizer temperature was 150°C and 450°C respectively.

### Fatty acid composition analysis

50 2-cell embryos were put into a tube, and 1ml chloroform-methanol (2:1, V/V) solution was added. After thoroughly homogenized, the tubes were stored overnight at -20°C. 0.3 ml 0.88% KCl was added to promote delamination and the chloroform phase was obtained and dried using N2. The transesterification was performed in 1ml 2% sulphuric acid-methanol (v/v) at 60°C for 1 hour, and later 2ml hexane and 0.5 ml saturated NaCl was added to promote the fatty acid ester extraction. Subsequently, the fatty acid ester extractions were washed 3 times with 2 ml H_2_O. Moreover, anhydrous sodium sulphate was used to absorb residual water. Ultimately, the hexane phase was obtained and dried using N_2_ and the fatty acid methyl ester was dissolved in 50μl acetone.

Fatty acid methyl esters were analyzed by Gas Chromatography-Mass Spectrometry (GC-MS) on a Thermo Focus GC/DSQII-MS with a hydrogen flame ionization detector and a HP-5-MS column (30 m×0.25mm×0.25μm). Helium served as the carrier gas, and 1μl sample was loaded when the injection temperature was 260°C. Column temperature program was set as follows: 140°C for 2min; 140°C to 170°C at 4°C/min, 170°C for 1min; 170°C to 240°C at 3.5°C/min, 240°C for 12.5min; 240°C to 260°C at 12°C/min. The detector temperature was 260°C [30]. Fatty acid methyl esters were determined by MS databases.

### Quantitative real time RT-PCR (qRT-PCR)

Total RNA from ovaries and embryos was extracted with Trizol (Invitrogen), and the cDNA was synthesized using purified total RNA with SYBR PrimeScriptRT-PCR Kit (TaKaRa). qRT-PCR were performed using the SYBR-Green I (TaKaRa) on ABI 7900 thermocycler. *Gapdh* served as an internal control for analyzing the gene expression level. The primer sequences for qRT-PCR were shown in Table 1.

**Table 1:** Primers designed for qRT-PCR.

GenBank Accession	Gene name	Sequence(5'to3')	Amplicon Size
NM_007988	*Fasn*	GGAGGTGGTGATAGCCGGTAT	140
		TGGGTAATCCATAGAGCCCAG	
NM_010719	*Lipe*	TCCTGGAACTAAGTGGACGCAAG	93
		CAGACACACTCCTGCGCATAGAC	
NM_011480	*Srebp1*	AGCCTGGCCATCTGTGAGAA	132
		CAGACTGGTACGGGCCACAA	
NM_010046	*Dgat1*	GCTCAGACAGTGGTTTCAGCAATTA	141
		ACAGAGACACCACCTGGATAGGA	
NM_026384	*Dgat2*	TGGCTACGTTGGCTGGTAACTTC	124
		GCATTGCCACTCCCATTCTTG	
NM_013523	*Fshr*	CCAAGATAGCAAGGTGACCGAGA	200
		CATGCAAGTTGGGTAGGTTGGA	
NM_013582	*Lhcgr*	CATTCAATGGGACGACGCTAATC	131
		GGCCTGCAATTTGGTGGAAG	
NM_008255	*Hmgcr*	TGTCCTTGATGGCAGCCTTG	143
		CCGCGCTTCAGTTCAGTGTC	
NM_008084	*Gapdh*	TGTGTCCGTCGTGGATCTGA	150
		TTGCTGTTGAAGTCGCAGGAG	
NM_001111274	*Igf1*	TGTGTAAACGACCCGGACCTA	109
		CTGGTGAAGGTGAGCAAGCA	
NM_001252658.1	*Ldlr*	AGTGGCCCCGAATCATTGAC	107
		CTAACTAAACACCAGACAGAGGC	
NM_016741	*Scarb1*	TTTGGAGTGGTAGTAAAAAGGGC	71
		TGACATCAGGGACTCAGAGTAG	

### Statistical analysis

SPSS 16.0 (SPSS Inc., Chicago, IL, USA) was used for the statistical evaluation. Each experiment above was repeated three times. Real-time PCR data was analyzed by 2^-ΔΔCT^ method. One-way ANOVA followed by a Fisher LSD post hoc test were programmed for the alterations among NC, LCOH and HCOH group, with the confidential interval of 95%.

## Results

### The number of MII oocytes and 2-cell embryos

We evaluated the number of MII oocytes and 2-cell embryos of 30 mice in NC, LCOH and HCOH group. The number of 2-cell embryos in LCOH group was significantly different from NC and HCOH group. The ovulation number showed no significant difference between LCOH and HCOH group (Table 2). These results indicated that high dose of gonadotropin might affect the fertilization rate.

**Table 2:** The number of MII oocytes and 2-cell embryo.

Group	NC	LCOH	HCOH
MII oocyte	10.93±3.11	20.92±4.07**	18.3±6.52**
2-cell embryo	10.71±2.91	20.30±4.18**	11.40±3.33^##^

Numbers are expressed as means ± SD. These results showed high dose of gonadotropin might decrease fertilization rate. Values with asterisks represent significant differences between the NC and COH group. Significant differences between HCOH and LCOH group are marked by hashtags.

**P<0.01

^##^P<0.01

### Ovarian weight index

The ovarian weight index was calculated using weight of the ovary (mg) divided by weight of the mouse (g) [31].

The ovarian weight index was significantly higher in LCOH and HCOH group compared with NC group, and no difference was found between LCOH and HCOH group at either 15h or 39h after hCG injection (Table 3). These results indicated that gonadotropin stimulation might have effect on ovarian weight.

**Table 3:** The ovarian weight index.

Group	NC	LCOH	HCOH
15h after HCG (%)	73.91±4.00	77.58±2.61**	81.67±6.45**
39h after HCG (%)	71.22±3.75	79.96±8.47**	83.31±2.47**

Numbers are expressed as the means ± SD. These results showed a gonadotropin induced up-regulation of ovarian weight. Values with asterisks represent significant differences between the NC and COH group.

**P<0.01

### The number of Secondary and antral follicles

**Fig 1:**
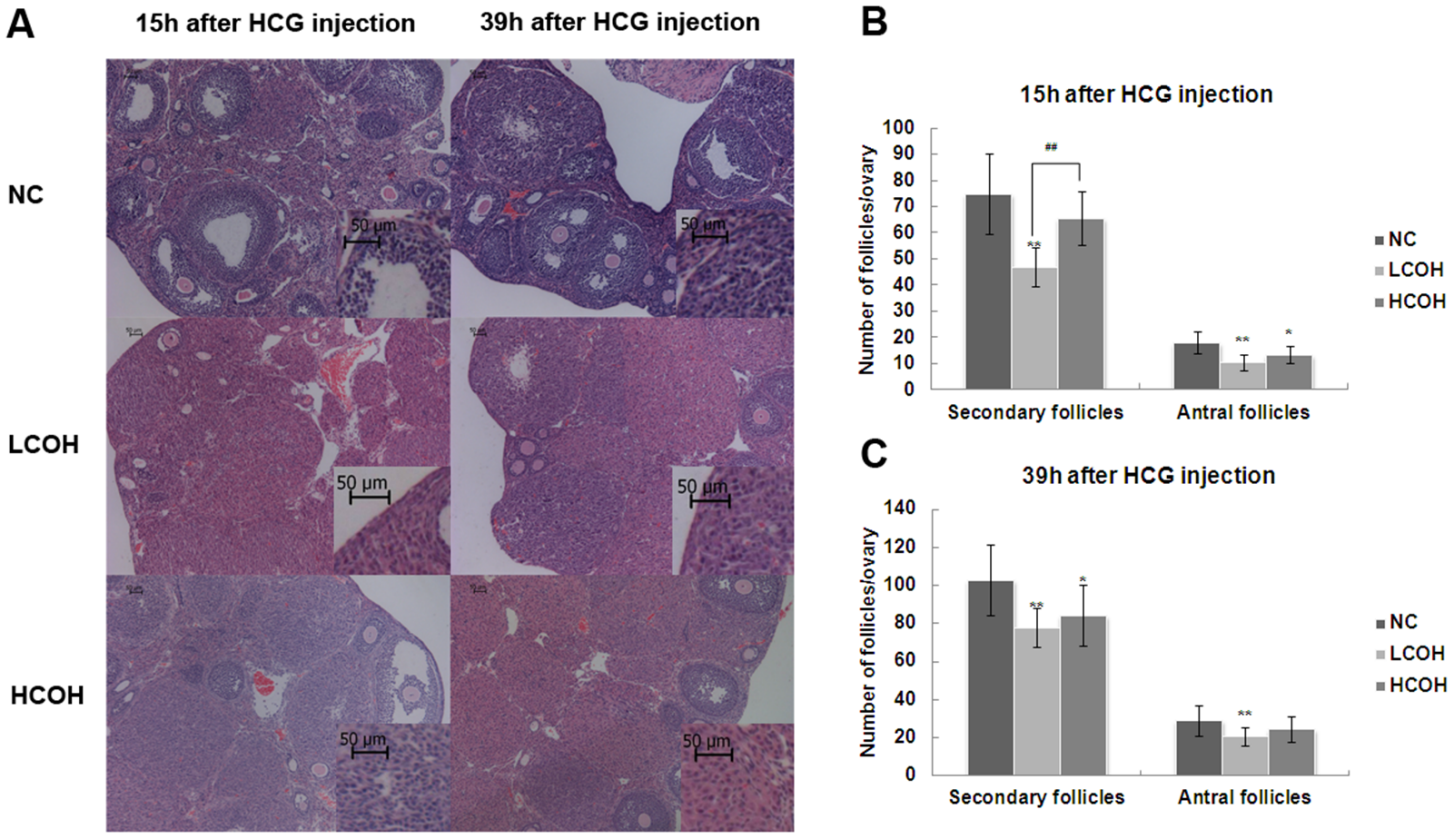
The number of secondary and antral follicles in ovaries of COH groups. Compared with NC group, corpus luteum was the main component in COH groups with fewer follicles (A). The comparison of secondary and antral follicles between NC and COH groups was shown in B and C. Values with asterisks represent significant differences between the NC and COH groups (* P<0.05, **P<0.01). Significant differences between HCOH and LCOH group are marked by hashtags (^##^P<0.01).

The ovaries from normal estrous cycles contained numbers of follicles of each stage and corpus luteum, while corpus luteum was the main component in ovaries of COH groups (Fig 1A). Compared with NC group, the number of secondary and antral follicles in ovaries was lower in COH groups either 15h or 39h after hCG injection. The number of secondary follicles in HCOH group was more than that in LCOH group especially at 15h after hCG injection (Fig 1B and 1C).

### The content of ovarian cholesterol and serum progesterone

**Fig 2:**
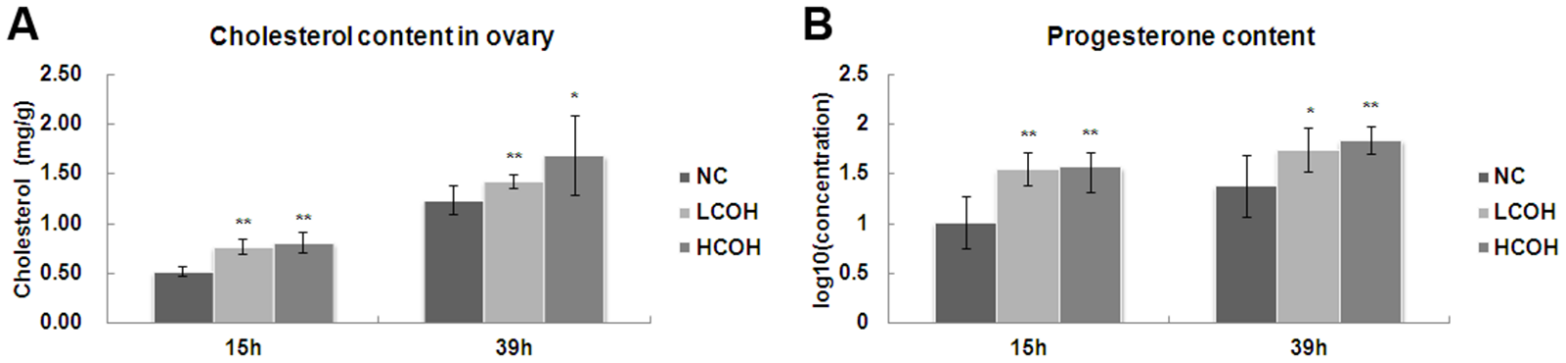
The content of ovarian cholesterol and serum progesterone. (A) The cholesterol content in ovaries of NC, LCOH and HCOH group; (B) The serum progesterone content in the three groups. Values with asterisks represent significant differences between the NC and COH groups (* P<0.05, **P<0.01).

The content of cholesterol (Fig 2A) and progesterone (Fig 2B) in two COH groups was significant higher than that in NC group. But no difference was shown between LCOH and HCOH group.

### The expression of gonadotropin-regulated genes in ovaries

**Fig 3:**
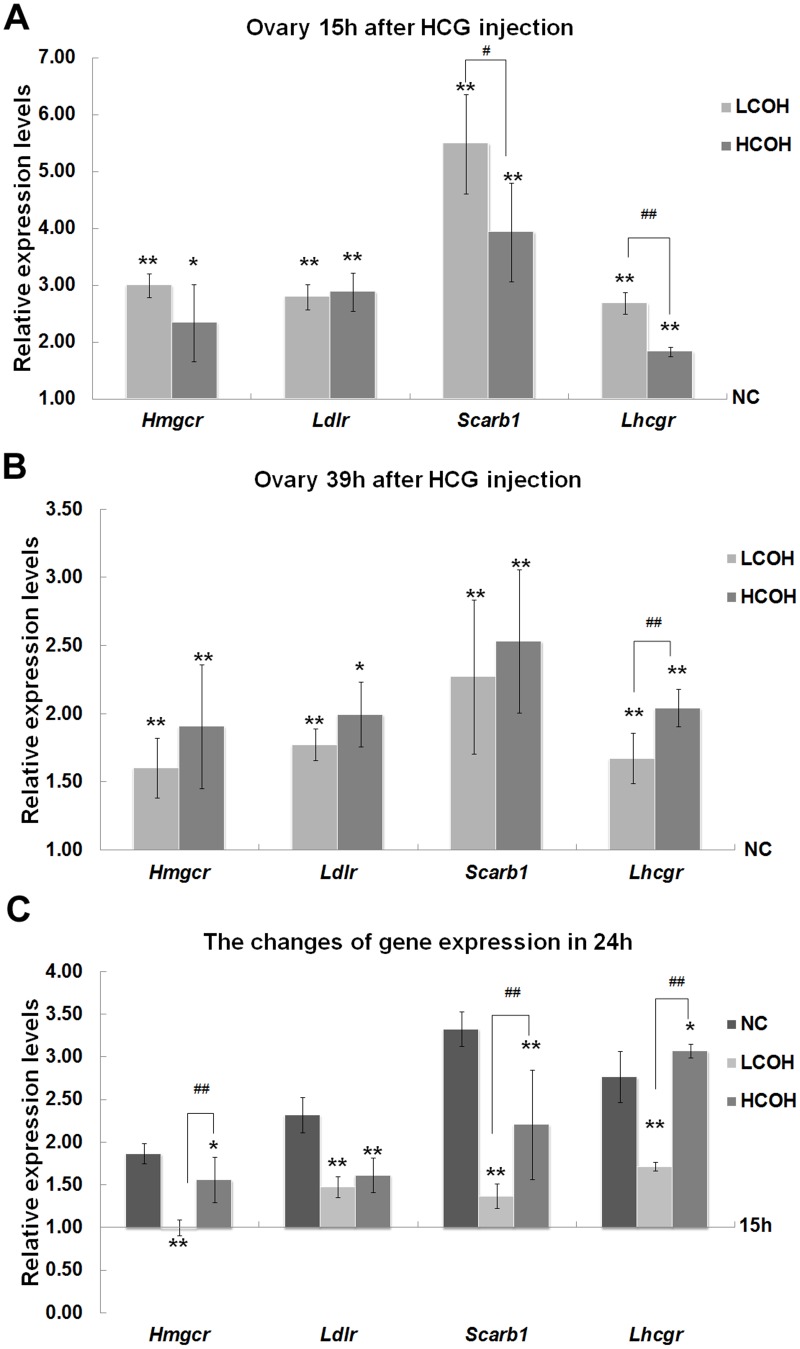
The expression of gonadotropin-regulated genes in ovaries. The expression of *Hmgcr*, *Ldlr*, *Scarb1* and *Lhcgr* between NC, LCOH and HCOH group at 39h- and 15h-post hCG injection was shown in A and B respectively. The expressed changes in the 24h presented in C. Values with asterisks represent significant differences between the NC and COH groups (* P<0.05, **P<0.01). Significant differences between HCOH and LCOH group are marked by hashtags (^#^ P<0.05, ^##^P<0.01).

The increased mRNA expression of HMG-CoA reductase (HMGCR), Scavenger receptor class B type I (SCARB1), low-density lipoprotein receptor (LDLR) and *Lhcgr* were shown in COH groups in ovaries 15h or 39h after hCG injection (Fig 3A and 3B). During the comparison between LCOH and HCOH group, the expression of *Scarb1* and *Lhcgr* was lower in HCOH group at 15h-post hCG injection, while *Lhcgr* was higher expressed in HCOH group at 39h-post hCG injection. The significantly different expression of these four genes was found in the ovaries between the two phases except *Hmgcr* in LCOH group (data were not shown). The expressed changes of *Hmgcr*, *Scarb1* and *Ldlr* in COH groups decreased with the control of NC group. The expressed changes of *Hmgcr*, *Scarb1* and *Lhcgr* in HCOH were significantly higher than LCOH group, and the change of *Hmgcr* in HCOH was even higher than NC group (Fig 3C).

### The lipid content in 2-cell embryos

**Fig 4:**
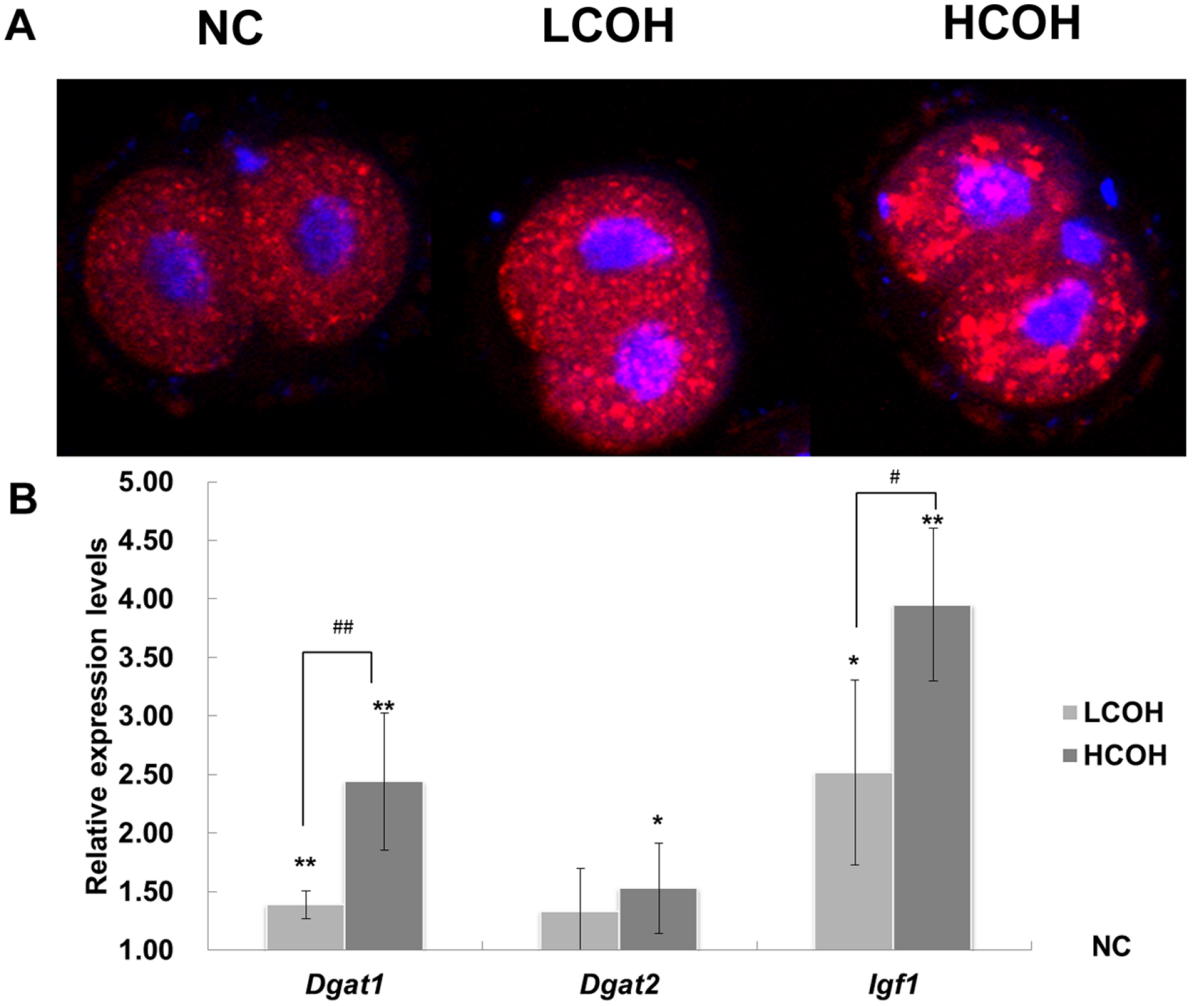
Lipid content in 2-cell embryos. The intensity of fluorescence in NC, LCOH and HCOH group increased progressively with gonadotropin dose (A). The expression of *Igf1* and *Dgat1* was also up-regulated in the similar pattern (B). Values with asterisks represent significant differences between the NC and COH groups (* P<0.05, **P<0.01). Significant differences between HCOH and LCOH group are marked by hashtags (^#^ P<0.05, ^##^P<0.01).

The lipid content and distribution was detected using Nile red. The lipid was characterized by aggregates in embryos, and lipid accumulation was gradually increased from NC, LCOH to HCOH group (Fig 4A). The mRNA expression of Insulin-like growth factor 1(IGF1) and diglyceride acyltransferases (DGATs) which closely co-relates with lipid accumulation was also increased in COH groups compared with the control NC group (Fig 4B). These results suggest that gonadotropin induced lipid accumulation in embryos in a dose-dependent manner.

### The expression of gene related to triglyceride metabolism in embryo

**Fig 5:**
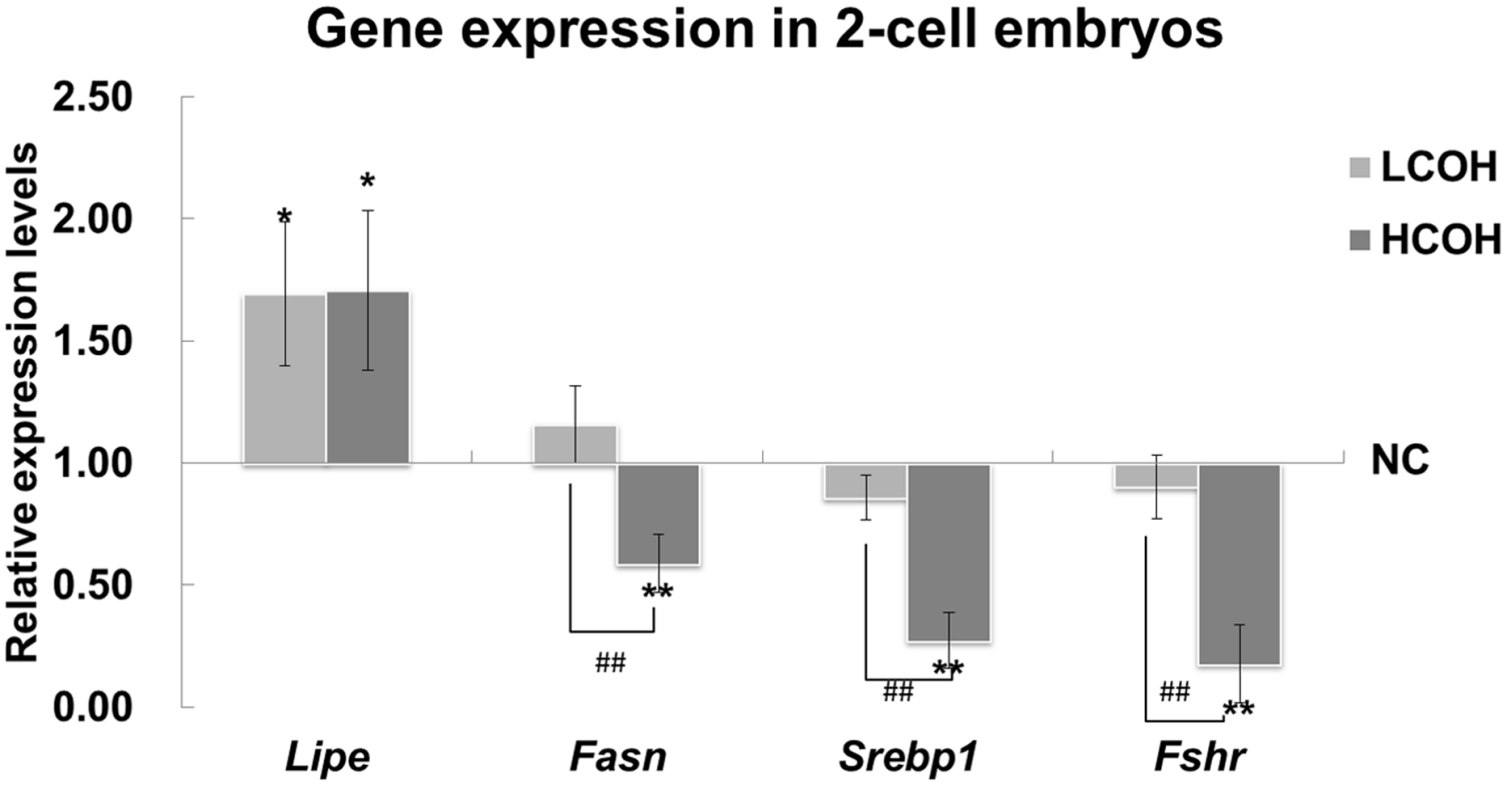
The expression of gene related to triglyceride metabolism in embryo. Compared with NC group, the expression of *Lipe* was up-regulated in COH group. The expression of *Fasn*, *Srebp1* and *Fshr* in HCOH group was lower than that in NC and LCOH group. Values with asterisks represent significant differences between the NC and COH groups (* P<0.05, **P<0.01). Significant differences between HCOH and LCOH group are marked by hashtags (^#^ P<0.05, ^##^P<0.01).

The mRNA expression of hormone-sensitive lipase (HSL/Lipe) in COH groups was higher than that in NC group. Significantly decreased mRNA expression of fatty acid synthase (FAS/Fasn), sterol regulatory element binding proteins1 (SREBP1) and *Fshr* was shown in HCOH group compared with NC and LCOH group, but there was no significant difference between NC and LCOH group (Fig 5).

### The fatty acid composition in embryos

In 2-cell embryos, 14 types of fatty acids were identified. In NC group, saturated fatty acids (SFAs) accounted for 75.93% of the total fatty acids, while monounsaturated fatty acids (MUFAs) and polyunsaturated fatty acids (PUFAs) had relative proportions of 17.84% and 6.23% respectively. Two predominant SFAs, palmitic acid (C16:0) and stearic acid (C18:0) accounted for 40.96% and 28.67%. The main difference among NC, LCOH and HCOH concentrated on the composition of 20-carbon fatty acids, especially arachidic acid (C20:0) and dihomo-γ-linolenic acid (DGLA, C20:3n-6). Compared with NC group, the content of C20:0 and C20:3n-6 had a gonadotropin dose-independent decrease. Moreover, Oleic acid (C18:1n-9) in LCOH group was significantly higher than NC and HCOH group. The content of Linelenic acid (C18:3n-6) in LCOH group was lower than that in the NC group. The details were shown in Table 4.

**Table 4:** The fatty acid composition in 2-cell embryos.

	NC	LCOH	HCOH
C14:0	4.80±0.65	4.15±0.01	4.49±0.25
C14:1n-2	0.11±0.08	0.14±0.01	0.14±0.05
C16:0	40.96±1.94	40.65±0.40	42.90±0.72
C16:1n-2	1.02±0.16	0.97±0.11	0.86±0.08
C16:1n-7	4.60±0.36	4.20±0.47	4.83±0.86
C16:1n-9	2.21±0.02	2.06±0.16	1.65±0.26
C18:0	28.67±1.85	29.11±0.76	29.58±1.87
C18:1n-9	8.31±1.31	9.97±0.16*	8.52±0.11^#^
C18:2n-6	3.09±0.29	3.73±0.87	2.83±0.05
C18:3n-6	0.57±0.09	0.36±0.02*	0.43±0.04
C20:0	1.50±0.03	1.12±0.16*	0.86±0.02**
C20:1n-9	1.59±0.76	1.51±1.02	1.06±0.45
C20:2n-6	1.42±0.21	1.34±0.09	1.32±0.06
C20:3n-6	1.16±0.21	0.70±0.02**	0.52±0.01** ^/^ ^#^
∑SFA	75.93±0.78	75.03±1.02	77.83±1.42
∑MUFA	17.84±0.41	18.85±0.10	17.07±1.49
∑PUFA	6.23±0.37	6.12±0.92	5.10±0.07

Numbers represented the percentage of each type of fatty acid. Data was expressed as the means ± SD. Values with asterisks represent significant differences between the NC and COH groups. Significant differences between HCOH and LCOH group are marked by hashtags.

* P<0.05

**P<0.01

^#^ P<0.05

## Discussion

In our research, superovulation was performed with two different gonadotropin doses. Between the two COH groups, the similar ovulation number but more 2-cell embryos in LCOH group indicated that high-dose gonadotropin may have negative effects on fertilization rate. The gonadotropin induced increase of mature follicles led to the alterations of weight and histomorphology in the ovaries after ovulation. Compared with LCOH group, the slightly increased secondary and beyond follicles in ovaries were found in HCOH group at both 15h and 39h after hCG injection. How did this occur?

hCG induced granulosa cell steroidogenesis are the main process during luteinization. The increased cholesterol in superovulated ovaries may result from varied expression of cholesterol-related genes induced by hormone. Although cholesterol can be produced by *de novo* biosynthesis, the major sources of cholesterol during luteinization derive from low-density lipoproteins (LDL) and high-density lipoproteins (HDL) through LDL receptor and the scavenger receptor BI (SR-BI) pathway [32]. Therefore, the expressions of the key enzymes during these three processes were detected. In our results, the accelerated increase of *Hmgcr*, *Ldlr*, *Scarb1* in COH groups was accompanied by the up-regulation of *Lhcgr*, which suggested the correlation between cholesterol and hormone. hCG induced depletion of ovarian cholesterol had been reported to be associated with the down-regulation of LHCGR[27] [33]. Our data supplied the direct evidences that the increasing cholesterol may result in the up-regulation of *Lhcgr* during corpus luteum phase.

The half-life of PMSG is 40–120h and hCG 24–36h respectively, while The half-lifes of FSH and LH are about 3h. Normal oestrous cycle which consists of ovarian follicle development, ovulation, and the formation and regression of the corpus luteum lasts for 4–5 days. Therefore, the gonadotropin levels of post-ovulation in COH groups were still high, especially in the HCOH group. The increased expressions of *Hmgcr*, *Ldlr*, *Scarb1* in superovulated ovaries suggested hCG might accelerate the luneinaization. However, we found that the expressions of *Hmgcr*, *Ldlr*, *Scarb1* in HCOH group were lower compared with those in LCOH group at 15h-post hCG injection. These data combined with the decreased histological changes of ovaries indicated that a long-time high level of gonadotropin may induce a long-time follicle development and ovulation which might inhibit luteinization and after 15h-post hCG injection, high dose of gonadotropin stimulation led to continuous high-rate increase of cholesterol.

Besides the effect of gonadotropin on the lipid metabolism in ovary, we also found the gonadotropin dose-dependent lipid accumulation in 2-cell embryos. Like previous observation, the lipid in embryos was characterized by aggregates of lipid droplets [34]. The Lipid accumulation was regulated by IGF1 in mesangial cell [35]. The LH/hCG signal can induce the increase of *Igf1* [36,37]. DGAT catalyzes the committed step in triglyceride synthesis. The up-regulation of both *Igf1* and *Dgat1* might interpret the lipid accumulation in embryos. Furthermore, increased progesterone can also induce lipid accumulation in human or rats [38,39]. Cholesterol is the precursor of progesterone, the increased progesterone induced by enhanced cholesterol in COH groups might also result in the lipid accumulation in embryos. Fatty acids are generally accepted as the energy provision during oocyte maturation and embryo development [25,26]. The inhibition of *de novo* lipogenesis has lethal effects on embryo development [40]. FAS is the key enzyme in *de novo* lipogenesis. In our results, gonadotropin decreased the fatty acid content in embryos by reducing the expression of *Fasn* and increasing the expression *Dgat1* and *Dgat2*. It was reported that the expression of *Fasn* and its transcription factor *Srebp1* was regulated by FSH [41]. However, hCG stimulation may inhibit FSH induced gene expression, and this effect sustained during the early stage of embryo development. Therefore, the decrease of fatty acids in embryos might be attributed to the remaining of internal high dose of hCG. In order to satisfy the demand of substrate for energy metabolism, *Lipe* was up-regulated to hydrolyze triglyceride to produce more fatty acids. The analysis of fatty acid composition of embryos showed the down-regulation of 20-carbon fatty acids in COH groups. DGLA is the precursor of Prostaglandin E1 (PGE1), which is an inhibitor of cholesterol synthesis [42,43]. The regulation of glucocorticoid on the sterol content is different from that of LH in ovary cycle. Glucocorticoid promotes steroid production before luteinization and inhibits it after luteinization. Glucocorticoid increases the production of cholesterol by blocking DGLA mobilization [44]. Therefore the reduced percentage of DGLA in embryo might originate from enhanced suppression of glucocorticoid to the high dose of hCG. Lipid accumulation induced by progesterone or enhanced IGF1 is positively associated with birth weight [38,39,45]. Different from the findings in human beings, our previous studies showed a higher birth weight in *in vitro* fertilization (IVF) or intracytoplasmic sperm injection (ICSI) mice models (N Wang, F Le & F Jin 2012, unpublished observations), and the same result was also found in preimplantation genetic diagnosis (PGD) mice model [41]. The variation of lipid metabolism in ovaries and embryos in our results also gave a potential warning of the alteration of birth weight.

## Conclusions

High dose of gonadotropin had effect on the quality of oocyte through increasing ovulation number and decreasing fertilization rate. The histological and morphological alteration of ovary accompanied by the changed mRNA expression of steroidogenesis-related genes suggested that gonadotropin might accelerate luteinization and lengthen the time of follicle development and ovulation. The lipid metabolism of oocytes was also affected by gonadotropin stimulation, and its effect sustained to 2-cell embryo stage. In embryos, the lipid accumulation was increased in a gonadotropin dose-dependent manner, while the fatty acid synthesis and 20-carbon fatty acids content were decreased. All the changes of lipid metabolism may have a potential influence on the birth weight of ART offspring. Whether the alterations of lipid metabolism induced by gonadotropin have influence on the growth of offspring needs further investigation.
